# Revisiting non-linear Thomson scattering with metamaterials

**DOI:** 10.1093/nsr/nwad230

**Published:** 2023-11-22

**Authors:** Natalia M Litchinitser

**Affiliations:** Department of Electrical and Computer Engineering, Duke University, USA

Non-linear optics is a branch of optics that studies the intriguing and sometimes unexpected ways in which light and matter interact at high intensities, when the polarization density does not respond linearly to the electric field of the light. The pursuit of the perfect non-linear optical material has been ongoing ever since the pioneering experiment on second harmonic generation carried out by Franken in 1961 [1]. Indeed, non-linear optical materials are of immense importance due to their unique ability to manipulate and control light in ways that linear materials cannot. They are crucial for devices and applications, such as frequency converters, optical modulators, switches, parametric amplifiers and laser systems, quantum information processing and biomedical imaging.

Non-linear phenomena typically rely on the inherent characteristics of particular materials (such as non-linear susceptibility) or on how much a material's properties change in response to the intensity of incident light, crystalline structure, bandgap and resonances [2]. For instance, only non-centrosymmetric crystals—those lacking an inversion center—support strong second-harmonic generation and other second-order non-linear processes due to the symmetry of their lattice arrangement. While various material systems that possess relatively robust quadratic or cubic non-linear responses have been discovered, a key challenge remains in combining the advantages presented by both non-linear optical materials and complementary metal-oxide-semiconductor electronics [3]. Moreover, the non-linear response is typically limited to a certain frequency range. In particular, the terahertz domain, which serves as a crucial link between electronics and optics, remains relatively underdeveloped with respect to non-linear optical materials [4].

In their recent research, Wen and co-authors present a unique approach to achieving effective non-linear responses through exciting non-linear Thomson scattering [5]. Non-linear Thomson scattering is a fundamental physical process in which charged particles, usually electrons, undergo non-linear oscillations when exposed to an intense electromagnetic field, leading to the emission of radiation at frequencies that are harmonics of the incident electromagnetic wave frequency [6]. Importantly, this phenomenon applies across the entire electromagnetic spectrum and is independent of symmetry. Nonetheless, for years the inherent weakness of non-linear Thomson scattering and the relatively weak magnetic contribution to the Lorentz force rendered it inconsequential in the context of natural solids.

The authors predicted and demonstrated that the synergy between a metamaterial split-ring resonator and a semiconductor where optical radiation triggers impact ionization (as shown in Fig. 1) gives rise to the second-order non-linear response within a centrosymmetric material. This new regime of light–matter interaction is due to the locally enhanced magnetic field and its interaction with charged carriers, inducing powerful non-linear Thomson scattering at moderate incident intensities and enabling very efficient non-linear responses in solids, such as single crystalline silicon, doped silicon and sapphire, which are independent of the symmetry properties of materials or the operating frequency.

**Figure 1.**
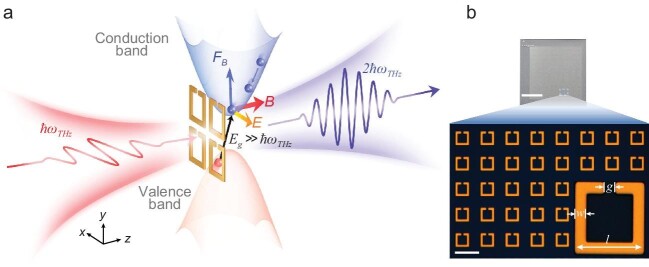
(a) A universal route to efficient non-linear response enabled by the synergy of non-linear Thomson scattering and local enhancement of electromagnetic fields in metamaterials. (b) The optical and microscopic photos of the metamaterials. Scale bars: (Upper) 50 mm; (Lower) 50 μm. Reproduced from Ref. [5].

In summary, this timely research article demonstrates a fundamentally new regime of non-linear responses in integrated terahertz systems with an orders-of-magnitude reduction in pump intensity, offering a new route to developing novel light sources in the terahertz spectral range and beyond.

***Conflict of interest statement*.** None declared.

## References

[1] Franken PA , Hill AE, Peters CW et al. Phys Rev Lett 1961; 7: 118–9. 10.1103/PhysRevLett.7.118

[2] Boyd RW. Nonlinear Optics, 3rd edn. Orlando, FL: Academic Press, Inc., 2008.

[3] Moss DJ , Morandotti R, Gaeta AL et al. Nat Photon 2013; 7: 597–607. 10.1038/nphoton.2013.183

[4] Lu Y , Zhang Q, Wu Q et al. Nat Commun 2021; 12: 3183. 10.1038/s41467-021-23526-w 34039972 PMC8155090

[5] Wen Y , Giorgianni F, Ilyakov I et al. Natl Sci Rev 2023; 10: nwad136. 10.1093/nsr/nwad136 37396487 PMC10313094

[6] Wegener M. Extreme Nonlinear Optics: An Introduction. Berlin: Springer, 2004.

